# Supplementary figures and images for: Learning poly-synaptic paths with traveling waves
Source: PLoS Comput Biol. 2021 Feb 9;17(2):e1008700. doi: 10.1371/journal.pcbi.1008700 (PMC7928500; doi:10.1371/journal.pcbi.1008700)

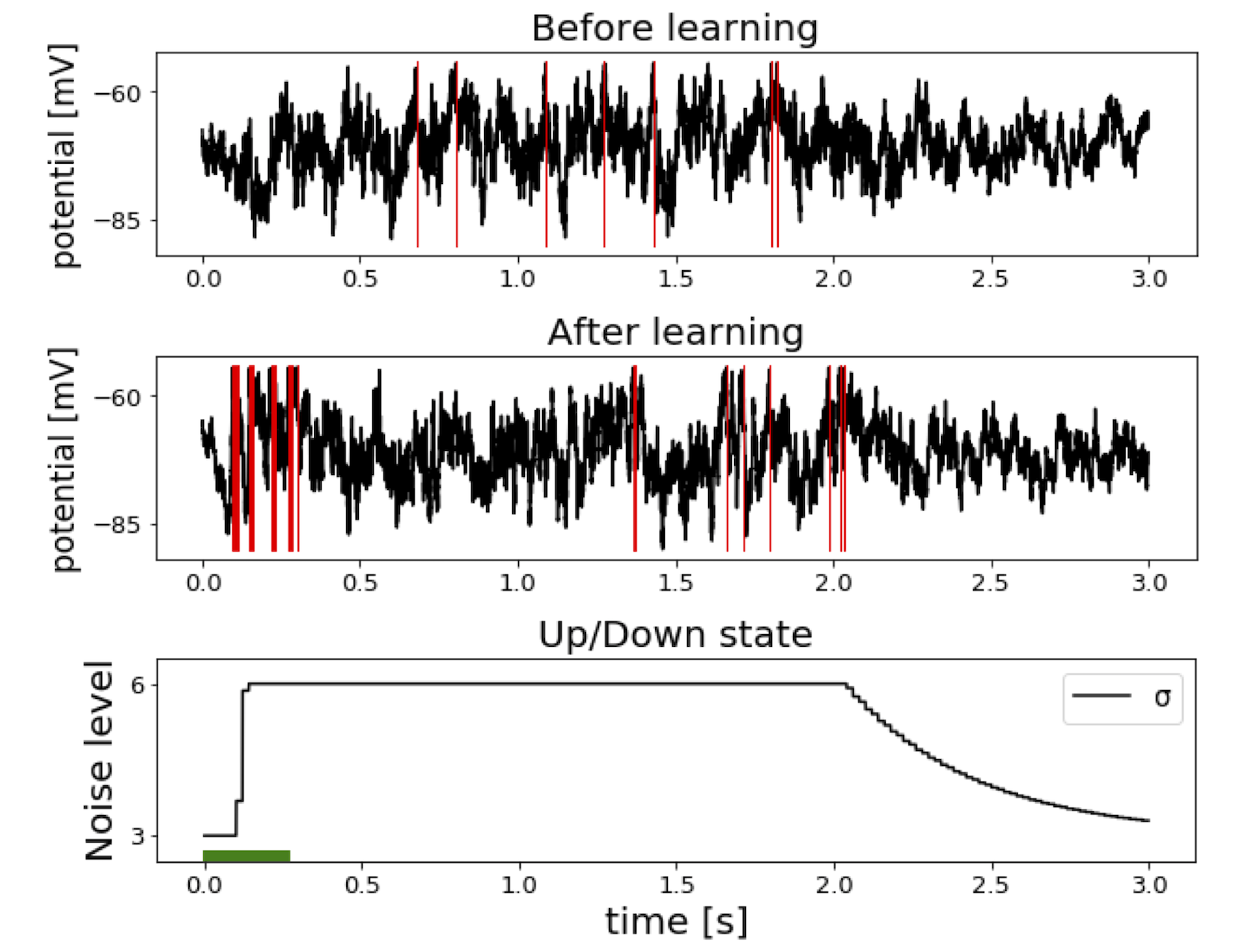

Supplement: S1 Fig — An example of the membrane potential before learning (Top) and the membrane potential of the same neuron after learning (Middle). The red line indicates spike timing. Before learning, the signal from the stimulated neuron does not reach the target neuron and the target neuron does not fire. In contrast, after learning, the external input reaches the target neuron, and the firing rate increases during the stimulus period. Meanwhile, the firing rate of spontaneous spikes driven by traveling wave does not change before and after learning. The noise level (black line) is changed by a traveling wave of upstate (Bottom). During the stimulus period at the onset of a trial, external input (green bar) is provided to the stimulated neuron. (TIFF) [file pcbi.1008700.s001.tiff]

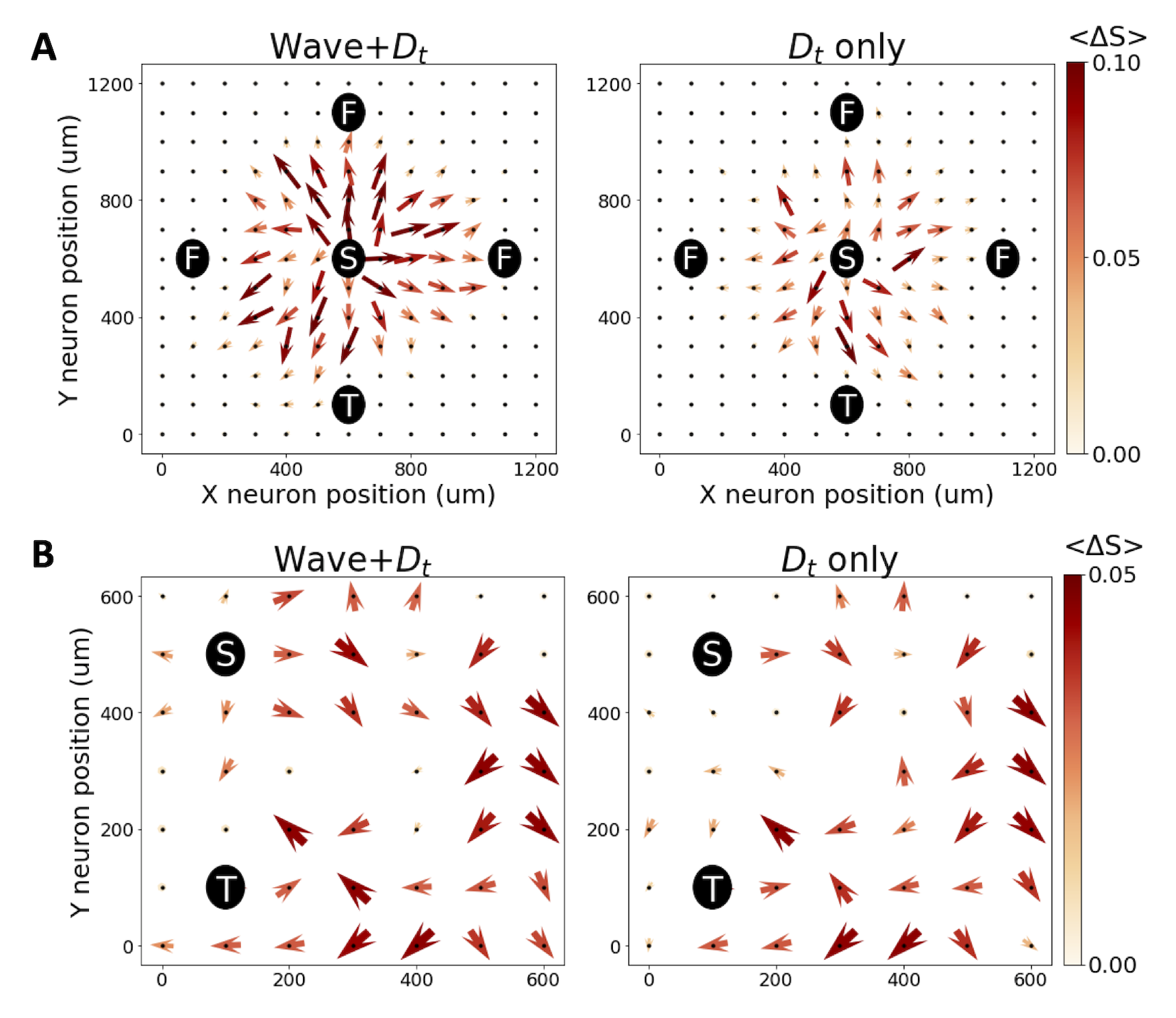

Supplement: S2 Fig — (A) The contribution of reward-independent STDP is shown for Task 1 by setting Dp = 0. The average synaptic weights are computed over 40 simulations, and their differences (from the initial trial to the 15th trial) are plotted with the Dt signaling and traveling waves (Left) and with the Dt signaling alone (Right). Outbound synaptic weights near the stimulated neuron are strengthened by the Dt signaling alone but more strongly with waves. In this task, initial synaptic weights are set rather strong. Hence, the stimulated neuron can propagate its activity to neighboring neurons from the beginning, and poly-synaptic paths toward the target and false-target neurons are gradually extended by reward-independent STDP. This happens even without waves but more efficiently with waves that contribute to the outbound spreading of neural activity. (B) The contribution of reward-independent STDP is shown for Task 2 by setting Dp = 0. The differences of averaged weights (from the initial trial to the 40th trial) are plotted with the Dt signaling and traveling waves (Left) and with the Dt signaling alone (Right). The detour path is efficiently strengthened in both cases because it is strong enough to propagate neural activity from the beginning. However, the shortcut path is strengthened only with waves because it is too weak to propagate neural activity at the beginning. Hence, the shortcut path requires waves to propagate neural activity only with waves, which is required to gradually strengthen the path by reward-independent STDP. (TIFF) [file pcbi.1008700.s002.tiff]
