# Supplementary material for: Learning poly-synaptic paths with traveling waves
Source: PLoS Comput Biol. 2021 Feb 9;17(2):e1008700. doi: 10.1371/journal.pcbi.1008700 (PMC7928500; doi:10.1371/journal.pcbi.1008700)
Supplement: S1 Text — (PDF) [file pcbi.1008700.s003.pdf]

Task 2 with feedforward network.

The same task as Task 2 was conducted with feedforward networks. Most of the setting is the same, but we changed the setting in the following three ways as follows:

- Adjacent neurons within 200 μm are unidirectionally connected. The directionality is determined based on the relative distance between the stimulated and target neurons. All connections are from a near-stimulated neuron to a near-target neuron.
- Because of the faster signal transmission compared to the recurrent network, we reduced the threshold of successful signal transmission latency from 100 ms to 75 ms.
- β = 0.025, d_{p} = 0.002, η = 2 are used for the best performance of this task.

The results of the feedforward networks (S1 Text Appendix A) is qualitatively the same as that of the recurrent networks (Fig 3). However, the contribution of D_{t} in the presence of waves is less evident in the feedforward setting. This indicates that reward-independent STDP is more important in recurrent networks to strengthen the shortcut paths from the stimulated neuron to the target neuron (S1 Text Appendix B). This indicates the importance of learning the directionality of signal propagation in recurrent networks. The D_{t} signaling and waves together can selectively strengthen paths that are outbound from the stimulated neuron.

In addition, we also confirmed that a relatively large wave amplitude η is needed for recurrent networks. For this task to be completed, η = 2 was the best in feedforward networks, while η = 5 was the best in recurrent network. This result suggests that recurrent networks require a large wave amplitude because wave signals disperse faster.

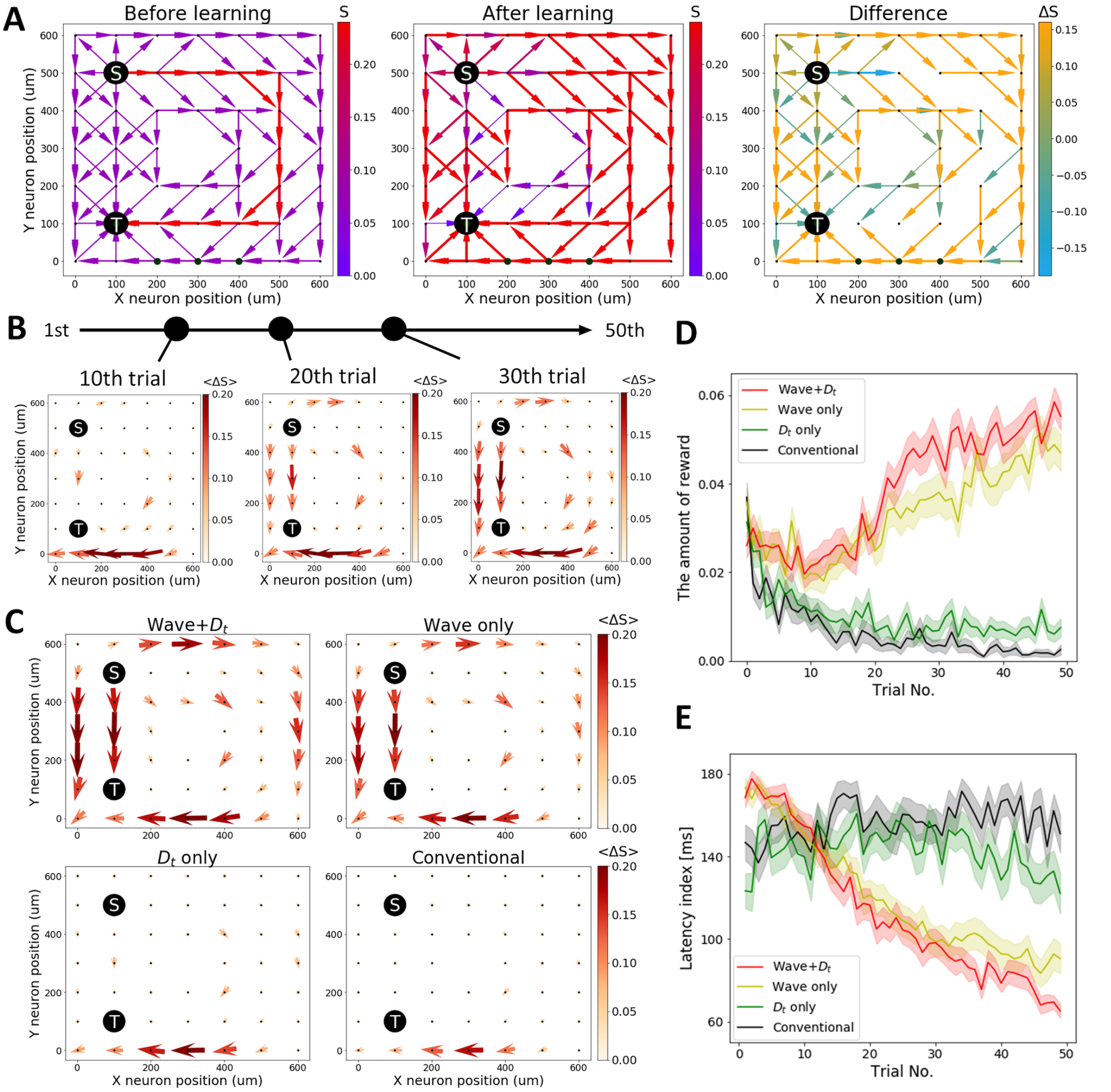

S1 Text Appendix A. Wave propagation helps to find a shortcut in feedforward networks. (A) A successful example of this task. Each panel represents the initial synaptic weight (Left), the last synaptic weight (Middle), and the difference between them (Right). Initially, weak short and strong detour paths from the stimulated neuron on the upper-left at S (100 μm, 500 μm) to the target neuron on the bottom-left at T (100 μm, 100 μm) are prepared. At the end of the trial, the short paths are strengthened while the detour paths are preserved. (B) The averaged synaptic weight difference from the initial trial to the 10th, 20th, and 30th trials is plotted. The averaged synaptic weights are calculated for each neuron, including the direction of synaptic connection. (C) The averaged synaptic weight difference from the initial trial and the last trial (the 50th trial) is plotted. (D) The amount of reward signal is plotted. The error bar indicates the standard error of

the mean. (E) The latency index takes the latency of the first spike in the target neuron after the stimulus onset if it is below 200 ms and takes 200 ms if the latency is above 200 ms. The condition with waves and tonic dopaminergic signal D_t (red) shows the best performance, while the conventional model (black) fails. The error bar indicates the standard error of the mean.

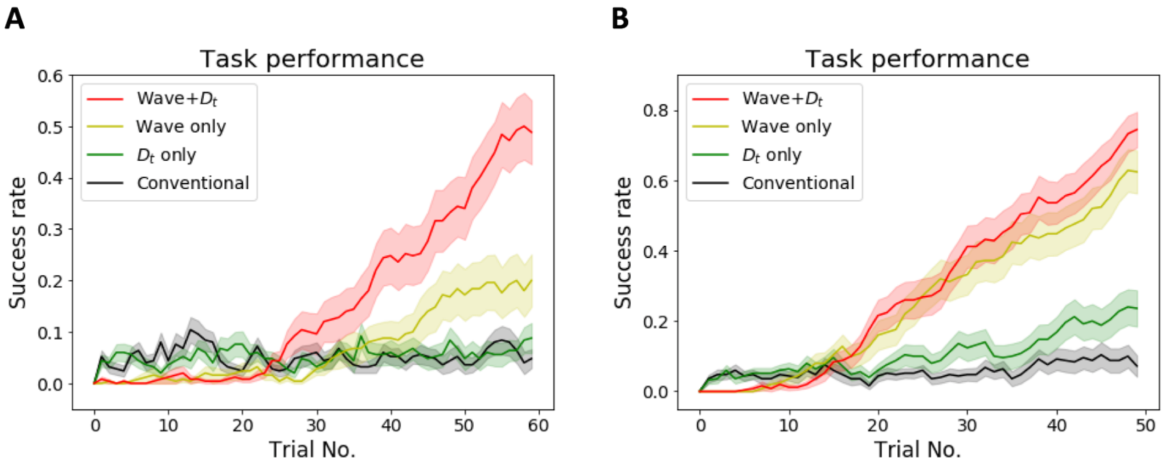

S1 Text Appendix B. The successful rate of Task 2.
(A) The result of recurrent networks in Fig 3. (B) The result of feedforward networks in S3 Fig. To evaluate the gradual maturity of the network, we defined the success rate as a 6-level step function. Under the restriction from 0 to 1, the success rate is added by 0.2 at correct trials while subtracted by 0.2 at incorrect trials. The correct trial is defined by the condition that the latency of the first spike in the target neuron after the onset of external input is less than 100 ms in the recurrent version and 75 ms in the feedforward version. These thresholds are chosen to have a near-zero success rate before learning. The definition of the successful rate corresponds to the value of $1 - Novelty$. The individual contributions of the D_t signal and waves are larger in the feedforward version than the recurrent version, and their effects are more synergistic in the recurrent version. The noise level of the wave-less models is set to a constant value that gives the same overall firing rate as the models with waves. This means that, before the arrival of a wave, the models with waves have a smaller noise level in the target neuron than the wave-less models. Consequently, the success rate of the wave-less models increases quickly by a small amount at the beginning because noise can occasionally cause short-latency spontaneous spikes in the target neuron and collect a reward.
