# Supplementary material for: Learning poly-synaptic paths with traveling waves
Source: PLoS Comput Biol. 2021 Feb 9;17(2):e1008700. doi: 10.1371/journal.pcbi.1008700 (PMC7928500; doi:10.1371/journal.pcbi.1008700)
Supplement: Attachment — Submitted filename: ToReviewer.pdf [file pcbi.1008700.s005.pdf]

Comments from Reviewer 1

The authors have presented modeling work that supports the hypothesis that waves in neuronal tissue facilitate memory consolidation. Their simple network model combines reinforcement learning through two-component dopamine modulation, local STDP, and excitatory waves of activity of a mean field coupled to local integrate-and-fire neurons. They show, through three different example tasks, that the waves depolarize broad swathes of neurons, allowing for better polysynaptic pathways to be found and reinforced through STDP and both tonic and phasic global dopamine modulation. Their model builds off of prior theoretical work that combined STDP and reinforcement learning using a single dopamine component.

I think this study is novel and of significant biological interest, uniting three different phenomena under the umbrellas of memory consolidation and computational efficiency.

We appreciate the referee for carefully reading the manuscript and finding the novelty and significance in our work.

My main concern is about the setup of the tasks that the authors might be able to address in a revision:

1) I was a bit unsure as to the biological motivation and context for the different network architectures. In contrast, the most direct preceding work (as referenced by the authors, Izhikevich 2007) studies a fairly realistic setup involving a randomly-connected sparse recurrent network with 80% excitatory and 20% inhibitory neurons representing L 2/3 in cortex. As it stands, in the present work there are three different architectures that require three different sets of parameters, including numbers of neurons, to perform the tasks well, and no suggestion as to the biological circuits in which they might be instantiated.

1a) The first, recurrent, network, which seems to have all-to-all nearest-neighbor connectivity for the non-stimulated and non-target neurons, seems the most plausible of the three. Should this be considered a part of a recurrent cortical layer as in Izhikevich?
1b) The second network is a simpler feedforward network. While feedforward networks may approximate the architecture in a cortical column or between different areas, it isn't clear if this second network matches these two contexts. Would it be possible to carry out this task within the recurrent network from task 1? If not, perhaps this can be mentioned as a current limitation, and suggested paths forward in this direction could be touched on in the discussion?
1c) The third network is a highly structured feedforward network. Given the spatial scale, should we consider this to be an approximation of elements from three different layers of a

cortical column? If not, providing a specific setting in which such a network might be instantiated would help motivate the biological plausibility of Task 3.

I believe the study, while interesting and novel as it stands, would be strengthened by addressing these points above.

We have added additional explanations about the biological motivation for using the specific network used in each task. For Task 1, we simulate a model of a cortical sheet, in which excitatory neurons are recurrently connected. We added the following sentences to the main text:

We use this recurrently connected neural network to model a two-dimensional cortical sheet. The task we consider is information routing in a cortical sheet required for some animal tasks, such as learning an appropriate action in response to a stimulus by preparing a path from visual neurons to motor neurons (Matsumoto et al., (2003)).

For Task 2, we previously used a feedforward network that the reviewer described. The motivation was to highlight the directionality of the signal flow in each path rather than based on biological plausibility. However, this feedforward assumption is not fundamental to the role of waves as well as the task performance. Therefore, we have moved this original simulation of the feedforward network to Supporting Information and added to the main text a new simulation using a recurrent network similar to what is used in Task 1 to solve the same Task 2. As expected, the results are qualitatively the same, indicating that our message about the role of waves in strengthening a shortcut path is robust.

For task3, we added the following sentence, explaining that the three-layer feedforward network may correspond to three different layers in the cortical column. Thank you for the suggestion.

We use a feedforward network in this task, which may be implemented for example in three information-processing layers (e.g., layer 4 to layer 2-3 to layer 5) in a cortical column (Alexandre et al. Neural Networks 1991; Lefort et al. 2008; Sakata and Harris, 2009; Jacob et al. 2017).

My more minor suggestions are:

i) While the results provided in the plots were described well and concisely, it would be nice to read a fuller examination that might suggest at least some further heuristic explanations for the results. Discussing these differences outlined below and what might account for them will help the reader to gain further insight into the author’s model:

ia) All three tasks show the "conventional" setup at or tied for the bottom of the four combinations tested. However, in Task 1, Dt was apparently an important isolated factor while waves seemingly lead to no increased success on their own, whereas in Tasks 2 and 3, waves seemed to contribute almost entirely to the increased success while Dt was far less important. Indeed, in Task 3, it is unclear if Dt helped at all, whether in isolation or in combination with waves, whereas it clearly provides a boost in Task 2.

To clearly show how the tonic dopamine signal Dt and waves help the network to learn the tasks, we added Figs. 2D, 3C, and 4C, which visualize how individual mechanisms help to shape the network. Moreover, to distinguish the contribution of reward-dependent and reward-independent STDP, we added Fig. S2 that shows the learning results without reward-dependent STDP. The roles of Dt and waves differ for different tasks because of their reward protocol and initial network setups, as we describe below.

For Task 1:

For this task to be completed, the D_t signal is critical because the input signal from the stimulated neuron does not reach the target neuron in the initial setting (Fig S1). Hence, a reward or punishment signal is too unreliable to train the network at the beginning. In contrast, reward-independent STDP, induced by the D_t signal, gradually establishes radially symmetric outbound paths spreading from the stimulated neuron (Fig S2). Traveling waves speed up this process by enhancing radially spreading neural activity, but they are not effective in the absence of the D_t signal because they drive noisy neural activity (Fig. S1). Once radially symmetric candidate paths are formed (Fig. S2), reward-modulated STDP can select paths toward the target neuron based on reward and punishment signals (Fig. 2).

For Task 2:

This network change occurs with a continuous reinforcement of shortcut paths (Fig 3B). The wave condition successfully establishes shortcut paths, while the no-wave condition cannot strengthen them (Fig 3C). The D_t signal enhances the role of waves by further strengthening the shortcut paths by reward-independent STDP but is not effective on its own because synapses along the shortcut paths are initially too weak to induce spiking activity in the absence of waves.

For Taks 3:

Fig 4D shows the task performance of each condition. The wave conditions (red and yellow) performs better than the non-wave conditions (green and black) because, similar to Task 2, the weak connections can only be strengthened with the support of traveling waves. However, the contribution of D_t is small in this task because the signal transmission from the stimulated neurons to the target neurons is easily achieved from the beginning in the presence of waves due to the dysynaptic feedforward structure.

ib) Less importantly, while waves do better eventually in Task 2, they seem to do worse for the first 15 trials. Is there a reason it takes this long for them to do well (this is not the case in Task 3)? Is there an obvious reason that the “conventional” condition increases its success rate very rapidly, but then stays essentially flat thereafter?

This effect is an artifact of the noise levels in the models with and without waves. In the models with noise, the noise level is dynamically regulated by waves. In contrast, in the models without waves, we use a constant intermediately-high noise level. This is explained in the main text as

To highlight the role of traveling waves, we also simulate models without waves. A constant noise level σ_i is used in these models. The value of σ_i is chosen so that the overall firing rate is the same as the corresponding model with waves.

In Task 2, a trial is successful when the latency of the first spike in the target neuron is less than a fixed threshold. Thus, the wave-less models tend to reinforce short-latency spontaneous spikes that are triggered by noise rather than by the shortcut path. To avoid the confusion due to the model artifact, we plotted instead the amount of reward and the latency index as new performance measures for they are more insensitive to the noise level. We moved the original figure about the success rate to Supporting Information, where the effect of noise is explained as follows:

The noise level of the wave-less models is set to a constant value that gives the same overall firing rate as the models with waves. This means that, before the arrival of a wave, the models with waves have a smaller noise level in the target neuron than the wave-less models. Consequently, the success rate of the wave-less models increases quickly by a small amount at the beginning because noise can occasionally cause short-latency spontaneous spikes in the target neuron and collect a reward.

ii) The “conventional model” does not seem to ever be defined, instead referring the reader to Izhikevich 2007 on line 200. I take it that it’s simply Dp with no Dt and no waves, in which case it would increase readability to define it here. It’s also a little unclear if this combination of Dp with no Dt and no waves can be so directly compared to the model in Izhikevich 2007 as to call it the “conventional model", given the large differences in architecture and model framework (e.g., all-to-all vs sparse connectivity, global inhibition vs. 20% inhibitory cells, ~100 neurons vs ~1,000).

Thank you for pointing out. As the reviewer pointed out, our focus is in the roles of waves and the learning rule, rather than in the network architecture. We used the term conventional model to indicate the Izhikevich model of “STDP”, which has no tonic dopamine signal Dt or traveling waves. To clarify the definition of the conventional model, we added the following explanations:

As a synaptic plasticity rule (Fig 1B, C), we used a modified version of reward-modulated STDP [26]. In this conventional model, synaptic plasticity does not occur in the absence of reward or punishment.

We set $D_t = 0$ for the conventional model.

To highlight the role of traveling waves, we also simulate models without waves. A constant noise level σ_i is used in these models. The value of σ_i is chosen so that the overall firing rate is the same as the corresponding model with waves. We define the conventional model as the model without the tonic dopamine signal and traveling waves.

iii) It would be helpful to explicitly define each network architecture and their initial conditions (initial weights), rather than just relying on the reader to inspect the graphs (which are a bit fuzzy). E.g., apart from the stimulated and target neurons for Task 1, is it a nearest-neighbor all-to-all network? Does the stimulated neuron connect only outwardly to all of its nearest neighbors (i.e., a nearest-neighbor source), and do the target neurons connect only inwardly to all of their nearest neighbors (i.e., nearest-neighbor sinks)?

Thank you for raising this pointing. We added an explanation of each network architecture in the main text and the corresponding captions:

Task 1: Synaptic connections from S are all outward, while the synaptic connections to T and F are all inward. Other neurons are randomly and unidirectionally connected to adjacent neurons within 200$\sqrt{2}$ μm with probability 0.5. If a neuron is isolated by chance, we repeat the procedure until it gets connected.

Task 2: Neurons within 100$\sqrt{5}$$\mu$m are randomly and unidirectionally connected with probability 0.5. If a neuron is isolated by chance, we repeat the procedure until it gets connected. The synaptic weights of a detour path are initially set three times as strong as the other synapses.

Task 3: In the initial setting, these middle-layer neurons receive a strong projection ($S_{ij} = 0.2$) from the nearest stimulated neuron and a week projection ($S_{ij} = 0.1$) from another randomly selected stimulated neuron. Two target neurons position at the top, namely, F (30 μm, 200 μm) and T (90 μm, 200 μm), and each middle layer neuron has a strong projection ($S_{ij} = 0.2$) to one of them.

iv) It's nice to see the examples in Figs 2A, 3A, and 4A. However, since the main results are averages over many realizations, might it also be helpful to show, perhaps in a middle row, the average of these realizations? Perhaps the averaged graph patterns will provide some additional useful perspective and intuition?

We agreed and added a figure of averaged weight to each task. For visualization, we calculated the average weight (including direction) for Task 1 (Fig. 2C) and 2 (Fig. 3B) to summarize the resulting network structures. For Task 3, we quantified the average weight of paths that transmit a specific input combination to show its contribution to the output (Fig. 4B).

v) Especially for Task 1, it seems there is substantial variation in the average performances (e.g., Fig 3C). It would be nice to have some handle on this variation, perhaps by providing shaded regions over the averages that represent the standard error over simulations for each condition?

Thank you for raising this point. We agree and added shaded areas representing standard error of mean in Figs. 2E, 3D-E, and 4D.

vi) Some additional description of Task 3 might be helpful for biologists without much of a CS background. A simple definition of XOR would be helpful, along with a description of how your task maps to this nonlinear function. To that end, some more intuitive neuron labels might be helpful. E.g., change (A,B,C,D) to (0_a, 0_b, 1_a, 1_b), and change (P, Q) to (T, F). In that case, (0_a, 1_a) and (0_b, 1_b) both map to T, and (0_a, 0_b) and (1_a, 1_b) both map to F, as one should expect for XOR.

Thank you for the useful suggestion. We changed the labels of inputs and outputs and updated Fig. 4 accordingly so that the mapping we use directly corresponds to the definition of the XOR gate. In the text, we describe:

During this task, four different stimuli are provided, where one of the pairs of stimulated neurons $0_a 0_b$*,* $0_a 1_b$*,* $1_a 0_b$*, or* $1_a 1_b$ *receives external input. […] The target neuron for each of the four stimuli is F, T, T, and F, respectively.*

vii) I'm a little confused by the inset in Fig 3C. It says that the plots represent averages over successful trials (line 249), and most values are above 100 ms. But line 230 defines a successful trial as one wherein the latency is less than 75 ms. Given the main plot of Fig 3C, it seems that this definition is a trial-to-trial metric. Perhaps I've missed something? Also, is there a reason that "Dt only" and "conventional", which also have a nonzero success rate after trial 0, are not included in the inset?

We apologize for the confusion. The meaning of "the successful trials" was all trials in a successful simulation where the latency became less than 75 ms at the end of the simulation (i.e., at the 50th trial). This definition was to minimize the influence of long latency outliers, but we agree that it was confusing. Hence, we now plot the latency index averaged over all trials instead:

Fig 3E represents the averaged latency index for obtaining reward after the trial onset. The latency index is equal to the latency of the first spike in the target neuron after the stimulus onset but saturates for latency above 300 ms to be insensitive to outliers. The latency index decreases faster in the wave conditions than in the no-wave conditions.

The nonzero successful rates of “Dt only” and “conventional” models are due to a noise-driven spontaneous spike in the target neuron, as we explained in response to the comment (ib). To avoid the confusion due to this model artifact, we plot “the amount of reward” instead and moved the original result of “Success rate” to Supporting Information, Fig. S4. Again, the following explanation is added in its caption:

The noise level of the wave-less models is set to a constant value that gives the same overall firing rate as the models with waves. This means that, before the arrival of a wave, the models with waves have a smaller noise level in the target neuron than the wave-less models. Consequently, the success rate of the wave-less models increases quickly by a small amount at the beginning because noise can occasionally cause short-latency spontaneous spikes in the target neuron and collect a reward.

viii) I think that the presentation of how the waves are generated would be easier to read if the mathematical formulation for the waves given by \psi_i (line 150) were presented at the same time as that of \alpha_i in Methods, in part because they have shared parameters that could be described all at once. Overall, I think readability would increase greatly if lines 147 - 160 were moved to Methods, where \alpha_i is described in detail (i.e., from the sentence that begins at the end of line 147 to the sentence that ends at the end of line 160).

Thank you for raising this point. We repeated a brief explanation of \psi in Methods.

ix) Dp, while more complicated, would be easier to digest quickly if its description had the same format as Dp and the other main variable presentations. Instead of describing it as a product of items in line 431 that were first explained, readability would be improved if it were first introduced in equation form, like the other main variables, on line 387, proceeded by descriptions of its constituent variables.

Thank you for raising this point. We agreed and added equations of Dp that describe its dynamics in Methods.

Comments from Reviewer 2

The authors have adopted a reward-based learning rule to learn distant paths between a stimulated and a target neuron in a spiking network. This mechanism involves traveling waves to facilitate communication between neurons and to find the shortest path between neurons to propagate the input stimulus. In three different tasks, they have shown an improvement compared to the model suggested in ref 26.

Thank you for reviewing our manuscript and summarizing our work.

However, there are several issues with the current report:

Major points:

1- The novelty of the work is not clear. Is it the involvement of the traveling wave in weight updates through the eligibility trace between neurons? The mechanism has not been clearly highlighted, and it is not comprehensive how the traveling waves contribute to learning. There are several designed equations with arbitrary parameters which their roles in training the network is not clear.

As described in the title and the abstract, the novelty of the work is the finding that traveling waves can facilitate reinforcement learning of poly-synaptic paths in neural networks. In particular, the following description in Author Summary answers the reviewer's question:

Information transmission among neurons in this large network is thought to be crucial for our behavior. For this to be achieved, multiple synaptic connections along a poly-synaptic network path must be adjusted coherently during learning. Because previously proposed reward-dependent synaptic plasticity rule requires coactivation of pre- and postsynaptic neurons, learning can fail if a subset of neurons along a distant network path is inactive at the beginning of learning. We suggest that traveling waves that are initiated at an information source can mitigate this problem.

We hypothesize a specific mechanism in the main text as:

We hypothesized that a critical role of traveling waves is to propagate neural activity in between distant indirectly connected neurons. […] Together with the standard reward-independent STDP (Dan & Poo 2006), traveling waves could gradually create a repertoire

of paths spreading from a wave-initiating site. Once such repertoire is prepared, neurons are coherently activated along the paths so that reward-modulated STDP could select a subset of these paths to perform a task. We simulate computational models of reward-modulated STDP to study if traveling waves enhance learning.

We have tested this hypothesis by simulating three tasks. First, waves can help to reinforce a path to a target when the target activation provides reward signaling (Task 1). Second, traveling waves can strengthen shortcut paths when target activation with short latency is rewarded (Task 2). Finally, waves can help to learn the XOR mapping in multi-layer feedforward neural networks (Task 3). To our knowledge, the role of traveling waves in reinforcing poly-synaptic paths has not been demonstrated in a neural network model.

The reviewer is correct that the mechanism has not clearly highlighted in the previous version. Our model has three major components to learn the tasks. (1) Reward-dependent STDP by phasic dopaminergic signaling. (2) Reward-independent STDP by tonic dopaminergic signaling. (3) Traveling waves, radially spreading neural activity from a stimulated neuron. We added Figs. 2D, 3C, 4C, and S2 that explicitly show how these components interact. These new results support our hypothesis described above.

Regarding the equation design and parameter values, we explained in the main text and Methods what each variable and parameter represents. As described above, we explicitly tested how the results change when the tonic dopamine signal, the phasic dopamine signal, traveling waves are removed. Especially, we carefully explained in Methods the equations for inhibitory feedback, dopaminergic signals, reward protocols, local field, and influx coefficients. Please, note that the way we model the dynamics of the variables is heavily constrained by the design of event-based simulation software Brian2.

In addition, we commented on the robustness of our model:

We use the same parameters (except three parameters summarized in Table 1) to learn three qualitatively different tasks in different network architectures, which underscores the robustness of the learning rule and the role of traveling waves.

Regarding the three setup-dependent parameters summarized in Table 1, we explained how the parameter values are chosen according to the network structure and the task in Table 1, and tested how the choice of parameters is dependent on the network architecture (recurrent networks vs. feedforward networks) in Supporting Information.

Inhibitory feedback strength β roughly correlates with the number of neuron N. Dopamine signal initial amplitude d_p is chosen for the best result for each task. Wave amplitude constant η is chosen depending on the network structure. Recurrent networks need relatively larger value compared to feedforward networks (see, Supporting Information, for solving Task 2 in feedforward networks). Note that, among several parameters in the model, β, d_p and η are chosen as representative parameters that control the basic ingredients of the model: global inhibitory signal, dopamine signal, and traveling waves, respectively.

2- As the authors have pointed out in the Discussion, the time scale of learning and that of the traveling wave that comes from neuronal activity are very different. Basically, the variable ψi could reflect any slow variable that relates neuronal activity to one another. Why do the authors call this variable "traveling wave"?

As the reviewer pointed out, the time scale of traveling waves is much slower than the feedforward propagation of spiking activity in our model. However, we did not write that the time scales of waves and the STDP time-window are very different–in fact, their time scales are overlapping. We modeled relatively slow traveling wave propagation (about 20 ms delay to next neurons (Sato et al. 2012) because the biological results suggest this. Grinvald et al., (1994) showed that it takes about 200 ms for the traveling wave to propagate in one cortical region, and Sato et al. (2012) showed that it takes about 100 ms to propagate in cortical layers. These results suggest that traveling wave propagates much slower than feedforward propagation of spikes as we model. Hence, we believe that the time scale we used for the traveling wave is biologically plausible. Although it is an interesting problem how the slow time scale of waves emerges from the network interaction of spiking activity, this will be a separate research topic.

We propose that such slow traveling wave propagation (about 20 ms delay to next neurons) is enough to trigger STDP (see, Bi & Poo 2001). Even if the effect of traveling waves in one trial is small, repetitive waves can gradually strengthen network paths, as we demonstrated in simulations.

3- How do the authors choose the initial conditions for equation in line 150, when the relation between ψi and the membrane potential is not clear? Is there a global minimum (unique solution for the weights) for any initial conditions?

Thank you for raising this point. As we explained in Results, local field ψ_i represents the average activity of a non-modeled neuron-mass around neuron *i*, and ψ_i controls the noise amplitude of neuron *i*. Without any external stimuli, ψ_i decays down to zero. We write:

As an initial condition, we choose $\psi_i = 0$ for all i, which corresponds to the downstate. We assume that upstate is induced by external stimuli (e.g., Tan et al., 2014).

We note, however, that this initial condition is not critical for our results. For example, randomly and sparsely activating some domain of ψ_i at the beginning of each trial does not significantly change our results because the trial duration 3 s is much longer than the decay time constant $200\ \mathrm{ms}$ of ψ_i.

4- Could the authors plot the input that a target neuron receives as a function of time (line 96), and compare the input that comes from the traveling wave, and the input that comes from the recurrent network dynamics due to spikes? It is important to see the relative

contribution of each input component.

Thank you for raising this point. First, while presynaptic spiking activity directly depolarizes membrane potential, traveling waves increase the amplitude of white noise added to membrane potential. Hence, their contributions to membrane potential are not directly comparable—we can instead compare their contributions to the output firing rate. We showed an example trajectory of the target neuron's membrane potential and spikes in Task 1 in Fig S1. Before learning, the spiking signal from the stimulated neuron hardly reaches the target neuron, but after learning, it sharply increases the target neuron's firing rate ~100 ms after the stimulus onset. Note that Fig 2B shows that a traveling wave can only increase its firing rate up to 5Hz, while its firing rate of successful cases increases up to 20 Hz. Thus, the increase of the target neuron's firing rate by spiking activity is much stronger and more phasic than that by a traveling wave after learning.

5- The roles of the tonic (Dt) and phasic (Dp) Dopamine signal are not clear. How differently do they contribute to learning, what makes the model significantly different that ref 26, and where does the improvement come from? Moreover, the authors used Dt+ Dp for the weight updates (line 126). In none of the equations in the text, Dp or Dt contribute independently/ individually to any other dynamics. Why the authors could not just consider one variable D which has the dynamics τp dD/dt = -D + k.Novelty(t), where k is a constant. The solutions for this equation upon choosing the right k can also give a linear combination of Dt and Dp that show up in line 126. In other words, what the authors have claimed in lines 302 – 306 has never been shown in the main text/ figures/ equations.

In our proposed model, Dt is always positive (it is almost a constant, just very slowly decays as the task-novelty declines toward the end of simulations), but Dp takes a positive value when a reward is provided and a negative value when punishment is provided, respectively. Thus, Dt induces reward-independent STDP, and Dp induces reward-dependent STDP. The total dopamine signal is described by the sum of the tonic signal (Dt) and the phasic signal (Dp). As the reviewer correctly summarized, only the sum Dt + Dp determines the magnitude and the sign of changes due to STDP. Both Dt and Dp signaling are modulated by novelty, as the reviewer pointed out. Hence, the variable D that the reviewer proposed is identical to our model (up to some minor change in the time-course due to the low-pass filtering of the novelty signal) if the coefficient k is a linear sum of a tonic component (kt) and phasic component (kp). Again, the tonic component kt should reward-independent, and the phasic component kp should be reward-dependent. We just explicitly described the kt-dependent dopamine signal as Dt and kp-dependent dopamine signal as Dp. This presentation is to highlight that the Izhikevich's STDP model is exclusively reward-dependent with only the Dp component (and Dt=0). Nonzero Dt allows the network to learn even without any reward or punishment signals.

Regarding the support for our hypothesis, we now have the following results on Task 1:

For this task to be completed, the D_t signal is critical because the input signal from the stimulated neuron does not reach the target neuron in the initial setting (Fig S1). Hence, a reward or punishment signal is too unreliable to train the network at the beginning. In contrast, reward-independent STDP, induced by the D_t signal, gradually establishes radially symmetric outbound paths spreading from the stimulated neuron (Fig S2). Traveling waves speed up this process by enhancing radially spreading neural activity, but they are not effective in the absence of the D_t signal because they drive noisy neural activity (Fig. S1). Once radially symmetric candidate paths are formed (Fig. S2), reward-modulated STDP can select paths toward the target neuron based on reward and punishment signals (Fig. 2).

6- How is the reward signal integrated in the STDP rule? In line 126, to update the synaptic efficacy, no STDP was used. It is not clear how figure 1B (Hebbian STDP) comes to the picture for synaptic updates.

How the reward signal is integrated into the STDP rule is the major focus of Izhikevich (2007), which is studied as the conventional model in our work. First, the STDP eligibility trace is explained as follows:

c_{ij} is so-called the STDP eligibility trace [26] that accumulates the effects of plasticity events with time-constant $\tau_c = 1000\,\mathrm{ms}$, […]. An increment of c_{ij} follows a typical asymmetric STDP window [34] with amplitude $\gamma = 0.0009$ and time-constant $\tau_{STDP} = 30\,ms$ (Fig 1B). The c_{ij} instantaneously increases if there is a pre-before-post event, instantaneously decreases if there is a post-before-pre event, and otherwise exponentially decays with the time-constant τ_c.

The actual synaptic plasticity depends on both the eligibility trace and the dopamine signal as the equation, $\mathrm{d}S_{ij}/\mathrm{d}t = c_{ij}(D_\mathrm{t} + D_\mathrm{p})/\tau_s$, indicates. Essentially, the sign of synaptic-weight-change follows the sign of the eligibility trance c_{ij} if the total dopamine signal $D_\mathrm{t} + D_\mathrm{p}$ is positive. This case is similar to the standard STDP except that the plasticity is expediated as a reward signal increases D_p. Contrarily, when a strong punishment signal makes the total dopamine signal $D_\mathrm{t} + D_\mathrm{p}$ negative, the sign of synaptic-weight-change is flipped. In this case, a pre-before-post event, which classically potentiates synaptic weight, depresses synaptic weight.

7- For all the three tasks that the authors have investigated, the mechanisms that are essential for a successful training must be shown in a figure to provide an insight about the key point of the model. Currently, only the success rate as a function of trial numbers, and the reward signals have been shown. The way that weights evolve with time and how they relate to other variables is not clear.

Thank you for pointing this out. We added Figs. 2D, 3C, 4C, and S2 that explicitly show how the following three mechanisms contribute for the final network structure: (1) Reward-

dependent STDP by phasic dopaminergic signaling, (2) Reward-independent STDP by tonic dopaminergic signaling, and (3) Traveling waves that radially spread neural activity from a stimulated neuron. Furthermore, we added Fig. 2C and 3B to show how network structure evolves during learning in Tasks 1 and 2. For Task 3, we summarize the functional network structure in Fig 4B to show how the XOR function builds up.

Minor points:

1- “learning distant paths” that shows up in the title is not comprehensive. Do the authors mean strengthening certain connections?

Thank you for pointing out. We actually meant “poly-synaptic paths”. The wording has been fixed throughout the manuscript.

2- It is not clear at all how the traveling waves find the shortest path between neurons. Can the authors explain that in the text (in line 79) how a reward modulated STDP can choose a subset of these paths?

Thank you for pointing out. Figures 3B-C and S2 are added to demonstrate how waves can help to find the shortcut. (Please, also see our response to Major point 7 for mechanisms.) Notably, we do not claim that the reward-dependent STDP can choose the shortcut path over the detour path—the detour paths are also strengthened. To clarify, we describe in the main text:

In Task 2, we show that traveling waves can empower reward-dependent STDP to reinforce initially weak shortcut paths.

Finding an initially weak shortcut path might be difficult without traveling waves because the neurons along the shortcut path would be seldom activated coherently. The wave propagation can significantly increase this probability and accelerate the learning process.

In a successful case, shorter paths are strengthened while the detour paths are moderately strengthened (Fig 3A).

3- Is there any general rule/ insight as to how to change η in the definition of the traveling waves to accommodate for different tasks?

The proper η value depends on the network structure. Recurrent networks need a relatively strong source to send waves down to distant neurons than feedforward networks. We confirmed this by comparing feedforward networks and recurrent networks in Task 2. Recurrent networks work best with relatively large η value ($\eta = 5$), while

feedforward networks work best with relatively small η value (η = 2). We added explanations of parameter tuning in the caption of Table 1 and Supporting Information.

4- In figure 5, there are two different update rules for Dp (one in discrete time the other in continuous time). These two are not consistent. Maybe in the figure caption this has to be explained to avoid confusion.

Thank you for pointing this out. These equations are consistent. Dp continuously decays in the absence of target (or false-target) neuron's spike but instantaneously jumps when the target (or false-target) neuron spikes. To avoid this confusion, we explicitly explained this in Methods:

D_p exponentially decays according to

$$\mathrm{d}D_\mathrm{p}(t)/\mathrm{d}t = -D_\mathrm{p}(t)/\tau_\mathrm{p}$$

except when a target / false-target neuron spikes. When a target / false-target neuron spikes at time t, D_p instantaneously jumps at time $t + t_\mathrm{p}$ according to

$$D_\mathrm{p}(t + t_\mathrm{p}) \mathrel{+}= R(t) \cdot \Gamma_R(t) \cdot \mathrm{Novelty}(t)$$

where decay constant $\tau_\mathrm{p} = 200\ \mathrm{ms}$, and transmission delay $t_\mathrm{p} = 100\ \mathrm{ms}$.

5- The references to some journals are not consistent in the Reference section (e.g. Journal of Neuroscience,…)

Thank you for pointing out. We fixed References.

6- Some sentences are not clear in the text. Better and more clear explanations are needed.

We added extra figures and sentences to clarify our points.

7- Figure fonts and resolution have to improve.

We improved figure fonts and resolution.
